# Effect of Doxycycline Microencapsulation on Buccal Films: Stability, Mucoadhesion and In Vitro Drug Release

**DOI:** 10.3390/gels7020051

**Published:** 2021-04-28

**Authors:** Venu Gopal Reddy Patlolla, Nikolina Popovic, William Peter Holbrook, Thordis Kristmundsdottir, Sveinbjörn Gizurarson

**Affiliations:** 1 Faculty of Pharmaceutical Sciences, University of Iceland, Hofsvallagata 53, 107 Reykjavik, Iceland; vgr1@hi.is (V.G.R.P.); nip2@hi.is (N.P.); thordisk@hi.is (T.K.); 2 Faculty of Odontology, University of Iceland, Vatnsmýrarveg 16, 101 Reykjavík, Iceland; phol@hi.is; 3 Costco Pharmacy, Kauptúni 3, 210 Garðabær, Iceland; 4 Pharmacy Department, College of Medicine, University of Malawi, Blantyre 3, Malawi

**Keywords:** doxycycline, matrix metalloproteinase inhibitor, MMPI, microparticles, film

## Abstract

The aim of this work was to stabilize doxycycline in mucoadhesive buccal films at room temperature (25 °C). Since doxycycline is susceptible to degradation such as oxidation and epimerization, tablets are currently the only formulation that can keep the drug fully stable at room temperature, while liquid formulations are limited to refrigerated conditions (4 °C). In this study, the aim was to make formulations containing subclinical (antibiotic) doxycycline concentration that can act as matrix metalloproteinase inhibitors (MMPI) and can be stored at temperatures such as 25 °C. Here, doxycycline was complexed with excipients using three techniques and entrapped into microparticles that were stored at 4 °C, 25 °C and 40 °C. Effect of addition of precomplexed doxycycline microparticles on films: stability mucoadhesion capacity, tensile strength, swelling index and in vitro release was studied. The complexation efficiency between drug-excipients, microparticles and films was studied using Fourier-transform infrared spectroscopy (FTIR) and differential scanning calorimetry (DSC). Two of the films were found to be stable at 4 °C but the film containing microparticle composed of precomplexed doxycycline with β-cyclodextrin, MgCl_2_, sodium thiosulfate, HPMC and Eudragit^®^ RS 12.5 was found to be stable at 25 °C until 26 weeks. The addition of microparticles to the films was found to reduce the mucoadhesive capacity, peak detachment force, tensile strength and elasticity, but improved the stability at room temperature.

## 1. Introduction

There is a need for topical drug delivery systems that can be used to treat oral conditions and is able to tolerate the harsh conditions inside the mouth. There is also a lack of commercially available formulations to treat the different conditions and diseases. When a product is developed that needs to stick to the mucosa for a certain period of time, we must understand the balance between therapeutic benefits and toxicological effects, such as irritation, etc. There is a clear benefit, that by treating such conditions locally, systemic side effects will be reduced as well as presystemic elimination of molecules [1].

The buccal mucosa is robust and tolerant to numerous compounds such as potential allergens [2] and is easily accessible for topical formulations. However, its unique physiology is challenging for all formulations that are placed in this environment, where there is a rapid salivary flow, effective mucus blanket layer and other mechanical movements that may damage or potentially eliminate the formulation [1]. Mucoadhesive buccal films are increasingly employed to deliver new drug candidates orally as they offer some unique advantages compared to hydrogels and buccal tablets. Some oral conditions could benefit from barrier properties of the buccal films, while compounds like doxycycline that acts as a matrix metalloproteinase (MMP) inhibitor may be delivered as well. The role of MMPs in conditions like aphthous ulcers [3], cold sores, oral mucositis [4] and periodontitis [5] is well known. Therefore, topical application of subclinical (antimicrobial) dose of doxycycline may be used to effectively downgrade the inflammation associated with these conditions [6,7,8]. Apart from MMP inhibition, these conditions require some barrier protective layer that could prevent further exacerbation from mechanical movements of the mouth.

Doxycycline is a very unstable compound which degrades and converts to its oxidation product metacycline, but also epimerization products such as 4-epidoxycyclline and 6-epidoxycycline that do not possess desirable pharmacological activity [9]. Oxidation may be caused by many factors such as exposure to water molecules, light or extreme temperatures. It can even occur at room temperature. It is, however, difficult to prevent epimerization of the compound. Studies have shown that preventing the compound from oxidation using antioxidants has been ineffective [10]. Hygroscopic materials or solvent remnants in buccal films could cause oxidation [11] and microparticles developed with hydrophobic polymers like Eudragit^®^ RS 12.5 could be beneficial in forming a barrier over doxycycline precomplexed with suitable excipients.

Buccal films are polymeric semisolid formulations, which can be custom made to any shape and are used to cover the affected site in the oral mucosa. The ideal film should be flexible, elastic, simulate the oral tissue and should not be fragile, while also offering the barrier layer over the affected surface. Chitosan offers good mucoadhesion by enabling the ionic interactions with the mucus glycoproteins [12,13] and it is also biocompatible, biodegradable, nontoxic and non-allergenic [14]. Chitosan is generally recognized as a safe (GRAS) additive by the United States Food and Drug Administration (FDA) for administration to the human body as well as a food additive or to be used in oral drug delivery [15]. Eudragit^®^ RS 12.5 offers some good extended release characteristics [16] but in combination with other excipients it may provide a unique blend of polymeric platform that is non-dissolving and an immediate drug release type which ideally can form a barrier jelly type layer over the effected surface. This could also reduce pain and aid in the healing process.

The main aim of this study was to find a formulation that can stabilize doxycycline in buccal films at 4 °C and 25 °C. In our previous work, doxycycline was stabilized in aqueous formulations [17]. One focus is to prevent epimerization from occurring by protecting the prone sites of doxycycline with suitable excipients in solid state, and then to encapsulate doxycycline with suitable polymers and incorporate the microparticles in buccal films. Degradation-sensitive compounds like doxycycline can be efficiently delivered but compatibility of polymers needs to be evaluated. Therefore, buccal films seemed to be an appropriate formulation strategy since they do not contain water molecules. It has been shown that Mg^2+^ ions, e.g., in the form of MgCl_2_ have been used to chelate the 4-N(CH_3_)_2_ site of doxycycline, and it showed beneficial effect on doxycycline stability [18,19]. Likewise, the antioxidant sodium thiosulfate has also shown beneficial effect on doxycycline stability [10]. Molecular modelling studies have also shown a beneficial effect of β-cyclodextrins on the stability of doxycycline where the aromatic ring or rings were completely encapsulated inside the hydrophobic cavity of β-cyclodextrins [20].

## 2. Results

### 2.1. Doxycycline and β-Cyclodextrin Complexation

**Figure 1:**
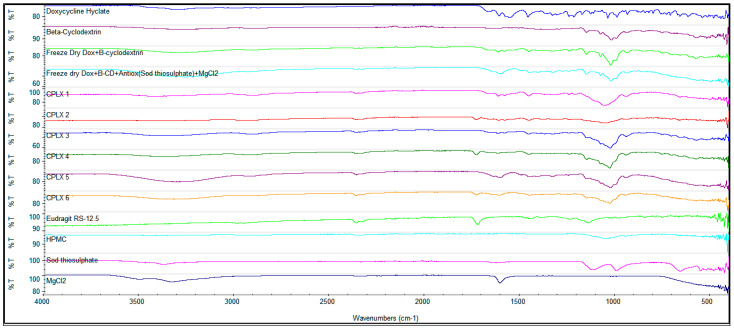
Fourier-transform infrared spectroscopy (FTIR) analysis of the following compounds. CPLX1 = doxycycline in 2% HPMC solution; CPLX2 = doxycycline in 2% HPMC solution with 0.25% Eudragit^®^ RS 12.5; CPLX3 = doxycycline and β-cyclodextrin in 2% HPMC solution; CPLX4 = doxycycline and β-cyclodextrin complex in 2% HPMC solution with 0.25% Eudragit^®^ RS 12.5; CPLX5 = doxycycline, β-cyclodextrin, sodium thiosulfate and MgCl_2_ complex in 2% HPMC; CPLX6 = doxycycline, β-cyclodextrin, sodium thiosulfate and MgCl_2_ in 2% HPMC with Eudragit^®^ RS 12.5; Eudragit^®^ RS 12.5; HPMC; sodium thiosulphate and magnesium chloride.

**Figure 2:**
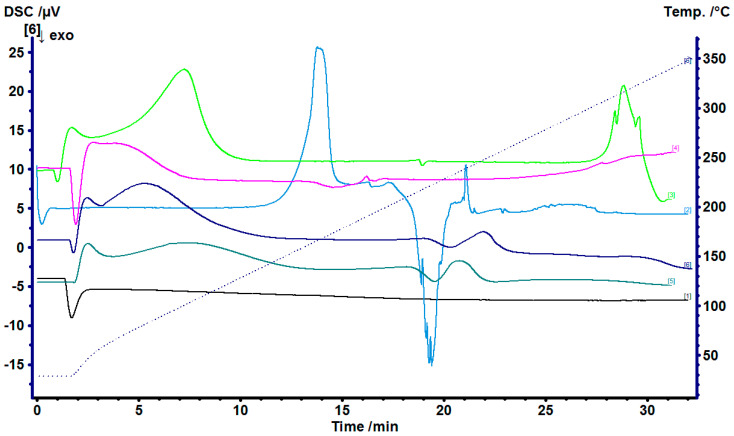
Differential scanning calorimetry (DSC) thermograms of: empty pan—black; pure doxycycline hyclate—light blue; pure β-cyclodextrin—light green; HPMC—pink; doxycycline-β-cyclodextrin (freeze-dried) + HPMC microparticles—green; and doxycycline-β-cyclodextrin in HPMC solution and HPMC microparticles—blue.

The doxycycline to β-cyclodextrin complexation was studied using Fourier-transform infrared spectroscopy (FTIR) and DSC (Figure 1 and Figure 2). The DSC peaks of doxycycline showed an endothermic peak at 170 °C and an exothermic peak at 230 °C. The pure β-cyclodextrin showed peaks at 90 °C and 325 °C. The exothermic peaks of doxycycline coincided with the physical mixture method and kneading complex indicating an uncomplexed doxycycline hyclate. The FTIR studies also showed the characteristic doxycycline peaks for the physical mixture method. The freeze-dried product showed good complexation for FTIR and DSC studies.

**Figure 3:**
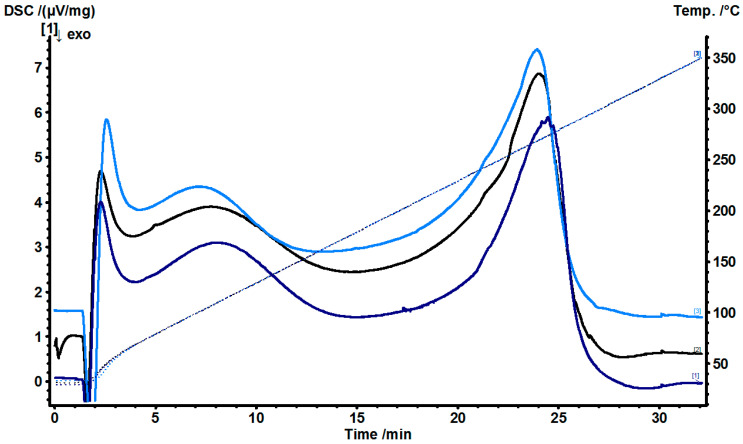
Differential scanning calorimetry (DSC) thermograms of Film 1 (bottom line) contained doxycycline in chitosan, gelatin, povidone, glycerin and Eudragit^®^ RS 12.5: Film 2 (middle line) contained doxycycline, β-cyclodextrin and HPMC microparticles in chitosan, gelatin, povidone, glycerin and Eudragit^®^ RS 12.5; and Film 3 (top line) was the same as in Film 2, but the manufacturing of microparticles was performed using sodium thiosulfate, MgCl_2_ in 2% HPMC with Eudragit^®^ RS 12.5.

The excipients used together with doxycycline in microparticles and films were also tested using the DSC as shown in Figure 2 and Figure 3. From Figure 3, the DSC thermograms of the Films 1, 2 and 3 were similar with slight peak shifts of endothermic peaks at around 280 °C, and none of the peaks corresponding to doxycycline or any excipient present showed indication of a formation of a new complex in the films.

**Figure 4:**
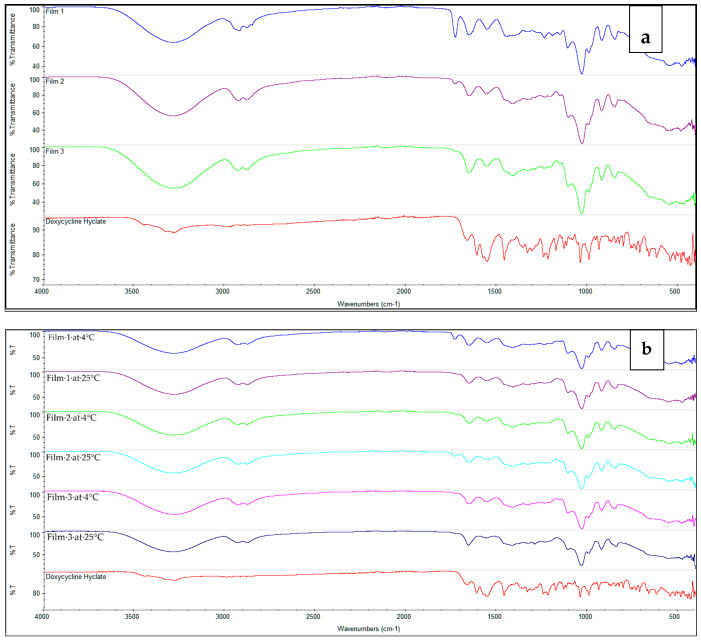
Fourier-transform infrared spectroscopy (FTIR) analysis of: pure doxycycline hyclate and in films at time zero (**a**) (top figure) and after 26-week storage at 4 °C and 25 °C (**b**) (bottom figure); Film 1 = contained doxycycline in chitosan, gelatin, povidone, glycerin and Eudragit^®^ RS 12.5; Film 2 = contained doxycycline, β-cyclodextrin and HPMC microparticles in chitosan, gelatin, povidone, glycerin and Eudragit^®^ RS 12.5; and Film 3 = same as in Film 2, but the manufacturing of microparticles was performed using sodium thiosulfate, MgCl_2_ in 2% HPMC with Eudragit^®^ RS 12.5.

The presence of Eudragit^®^ RS 12.5 in the microparticles gave a distinct peak at around 1700 cm^−1^ and MgCl_2_ gave a peak at 1600 cm^−1^. Sodium thiosulphate had a peak at 1000 cm^−1^ that disappeared during freeze drying together with doxycycline and β-cyclodextrin and MgCl_2_, indicating a new complex formation (Figure 1). The distinct Eudragit^®^ peak at 1700 cm^−1^ was only observed in Film 1, which indicates Eudragit in free form. Figure 4a shows the FTIR analysis of Films 1–3. The FTIR spectra were recorded for the films at 4 °C and 25 °C after 26 weeks (Figure 4b). Interestingly, the spectra for Film 3 almost remained unchanged at 25 °C.

### 2.2. In Vitro Mucoadhesion and Properties of the Buccal Films

**Figure 5:**
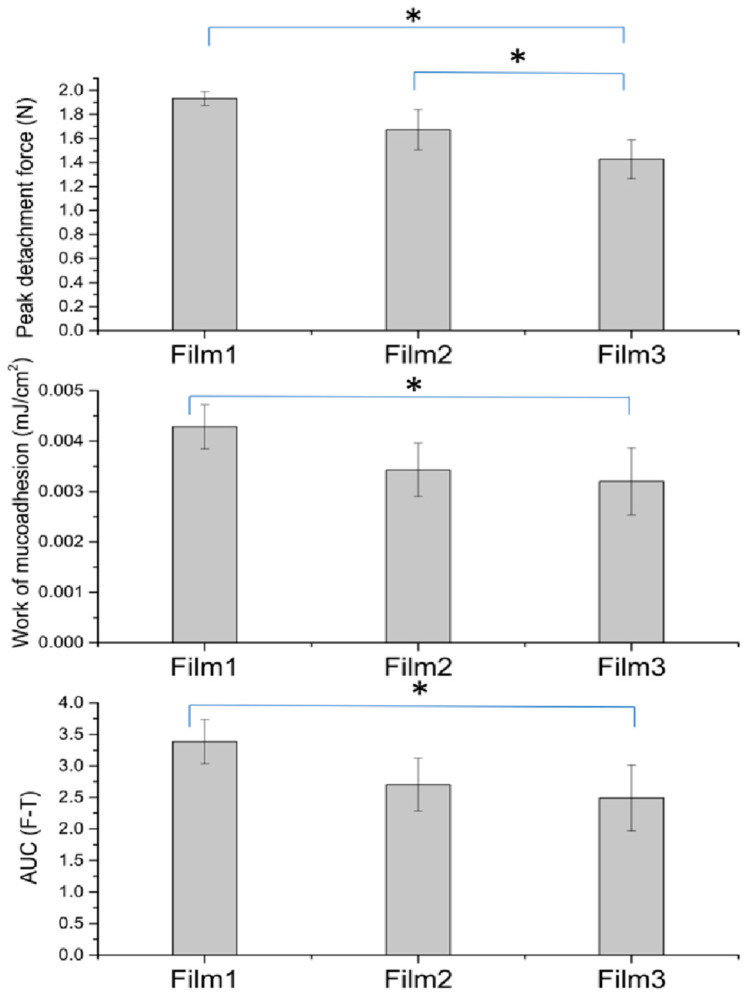
The peak detachment force, work of mucoadhesion and AUC (F-T) analysis of Films 1, 2 and 3, representing in vitro mucoadhesive evaluation (Film 1 = contained doxycycline in chitosan, gelatin, povidone, glycerin and Eudragit^®^ RS 12.5; Film 2 = contained doxycycline, β-cyclodextrin and HPMC microparticles in chitosan, gelatin, povidone, glycerin and Eudragit^®^ RS 12.5; and Film 3 = same as in Film 2, but the manufacturing of microparticles was performed using sodium thiosulfate, MgCl_2_ in 2% HPMC with Eudragit^®^ RS 12.5). * *p* < 0.05, one way ANOVA Turkey HSD post hoc test.

Addition of microparticles to the films significantly reduced the detachment force of the films as shown in Figure 5. The results were highly dose dependent since Film 3, which contained the highest density of microparticles, showed the lowest mucoadhesion. The work of mucoadhesion and area under force-time curve (AUC) also decreased in the presence of microparticles but the effect was not significant with the amount of microparticles incorporated (Film 3 and Film 2).

**Figure 6:**
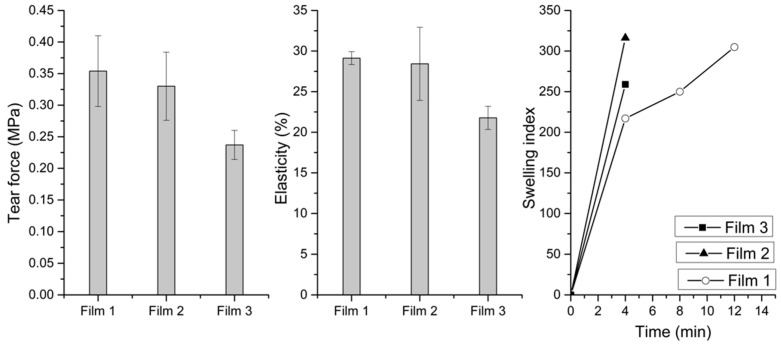
In vitro tensile strengths, elasticity and swelling index study of Films 1, 2 and 3.

The tensile strengths and elongation at breakage (elasticity) for the films are also shown in Figure 6. As for the mucoadhesion, increasing density of microparticles decreased both the tensile strengths and the elasticity of the films.

The swelling index of the films is shown in Figure 6, where the film containing doxycycline only showed slower water uptake compared with films containing microparticles. The swelling index, however, was similar for all the films, although the films containing microparticles swelled more rapidly. The thickness and pH on dispersion of these films were found to be as follows: Film 1: 0.3 mm and pH 4.24; Film 2: 0.33 mm and pH 4.57; Film 3: 0.38 mm and pH 4.83. The surface area of the films also more than doubled and the highest increase was observed for Film 1 and the lowest for Film 3.

### 2.3. Stability Studies

The stabilities of the microparticles at 4, 25 and 40 °C were found to be between 98–100% over a period of 6 months at all temperatures measured. The presence of Eudragit^®^ and β-cyclodextrin was found to impact the doxycycline stability.

**Figure 7:**
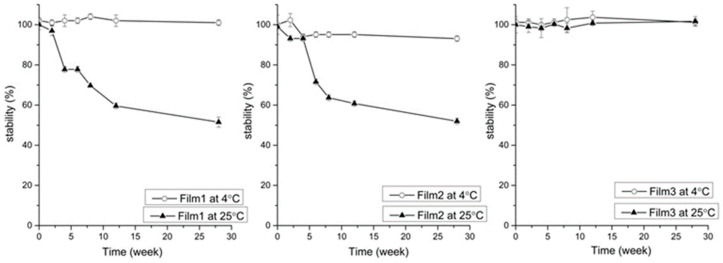
Stability of doxycycline in buccal films at 4 and 25 °C. Film 1 = contained doxycycline in chitosan, gelatin, povidone, glycerin and Eudragit^®^ RS 12.5; Film 2 = contained doxycycline, β-cyclodextrin and HPMC microparticles in chitosan, gelatin, povidone, glycerin and Eudragit^®^ RS 12.5; and Film 3 = same as in Film 2, but the manufacturing of microparticles was performed using sodium thiosulfate, MgCl_2_ in 2% HPMC with Eudragit^®^ RS 12.5.

The stability of the buccal films was also studied for 6 months at 4 and 25 °C. The film containing doxycycline only was found to be stable at 4 °C but became very unstable at 25 °C (Figure 7). The films containing microparticles and β-cyclodextrin complex with doxycycline showed decreased stability even at 4 °C. The presence of β-cyclodextrins did not show beneficial effect on the stability of doxycycline. The film containing antioxidants, chelating agent and β-cyclodextrins was found to improve the stability of doxycycline, even at 25 °C.

### 2.4. Microparticle Size

The microparticle size for all the complexes was found to be in the submicron range as follows: Complex 1 and Complex 2: 0.292 µm; Complex 3: 0.277 µm; Complex 4: 0.248 µm; Complex 5: 0.2 µm and Complex 6: 0.277 µm.

### 2.5. Dissolution and Release Studies

**Figure 8:**
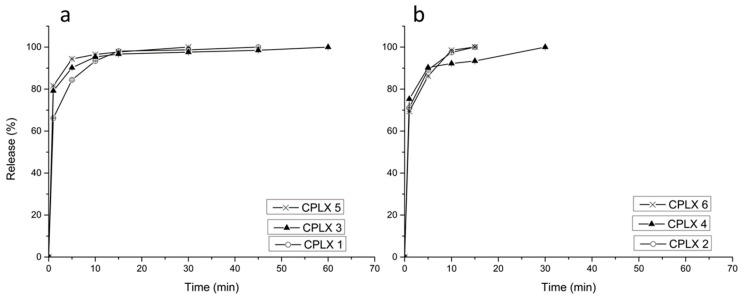
In vitro release of doxycycline from microparticles 1, 3 and 5 (**a**); and the same microparticles 2, 4 and 6 but containing additional Eudragit^®^ RS 12.5 (**b**). Microparticle 1 is doxycycline in 2% *w/v* HPMC solution; where microparticle 2 is the same as #1 but with 0.25% Eudragit^®^ RS 12.5; microparticle 3 is doxycycline and β-cyclodextrin in 2% HPMC solution; where #4 is the same as #3, but with additional 0.25% Eudragit^®^ RS 12.5; microparticle 5 is doxycycline, β-cyclodextrin, sodium thiosulfate and MgCl_2_ complex in 2% *w/v* HPMC; where #6 is the same as #5 but with additional 0.25% Eudragit^®^ RS 12.5.

**Figure 9:**
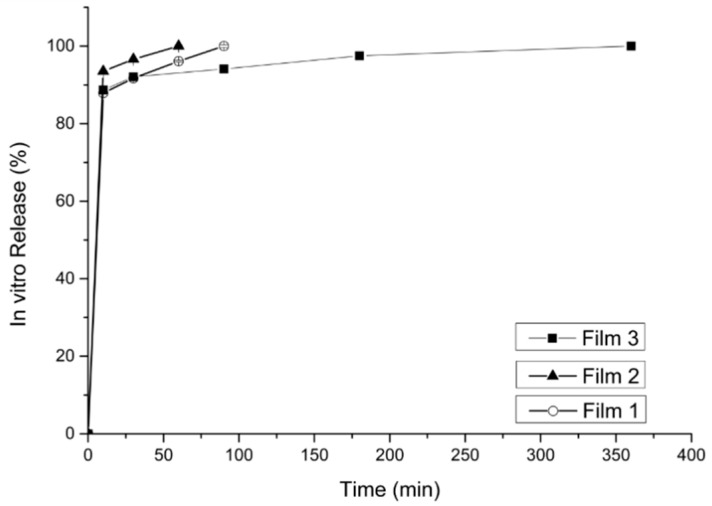
In vitro release of doxycycline from Films 1, 2 and 3.

The dissolution of doxycycline from the microparticles (Figure 8) showed that all formulations gave a rapid burst effect, releasing about 70% of the doxycycline within the first minutes, except formulations containing β-cyclodextrin (microparticles, 3 and 5) where 80% of the drug was released within the first minutes. The remaining drug was released within an hour. Eudragit^®^ has been used for controlled release, but in this study, it did not have an effect on the release. All the films (Figure 9) showed immediate burst release of doxycycline, where about 90% of the drug was released immediately.

## 3. Discussion

Doxycycline was found to be stable even at elevated temperatures at 25 °C and 40 °C. This shows that removal of water significantly improves stability compared with previous studies [18]. Even the semisolid formulations, buccal films, containing plasticizers and materials that may show hygroscopic characteristics, showed good stability of doxycycline. The buccal films were stored in desiccators that could prevent water from being absorbed into the films.

This study also shows that by adding precomplexed doxycycline microparticles to buccal films, most parameters were decreased, such as tensile strength, elasticity and mucoadhesion, but the swelling rate was improved. The swelling index and the surface area of the films doubled, slightly faster in the presence of microparticles, probably due to increased access to water from increased surface area of microparticles in films during the swelling process. The in vitro residence time was also shorter in the presence of microparticles. The FTIR and the DSC study confirmed that doxycycline had formed a complex, e.g., with β-cyclodextrin and MgCl_2_, thus improving the stability.

Both the microparticles as well as the buccal films presented a rapid burst effect, releasing between 70–90% of the drug within the first few minutes, followed by a slow release for the remaining doxycycline. The addition of Eudragit^®^ caused rapid drug release in all the complexes, but the presence of Eudragit^®^ was able to slow down the initial burst release from 80% to 70% for all the complexes.

Doxycycline was found to be fully stable in polymeric matrices consisting of chitosan, Eudragit^®^, gelatin, povidone and glycerin at 4 °C. Even though chitosan was used in 1% acetic acid solution, the acid did not seem to affect doxycycline’s stability. At 25 °C, the microparticles were well stabilized.

A minor peak of the epimerization degradation product, 4-epidoxycycline, was observed in Film 1 and Film 2 at 25 °C but the loss of doxycycline potency did not correlate with the small peak of degradant which might be due to secondary degradation of 4-epidoxycycline.

The burst release of drug even in the presence of Eudragit^®^ might probably be due to the submicron size of microparticles, which might cause inadequate entrapment in the polymer matrices. The burst effect can be beneficial in releasing doxycycline to the mucosa, affecting the MMPs. Optimally, it would have been better to have a gradient release over a longer period of time in order to continue inhibiting the MMPs. Since the dose of doxycycline is subclinical, it is necessary to achieve a certain burst effect to induce necessary MMP inhibition in the mucosa. This together with the swelling can be beneficial in forming a barrier over the inflamed sores/surface to prevent further exacerbation from mechanical movements from the mouth.

The addition of microparticles is promising in stabilizing doxycycline in solid and semisolid formulations, even at higher temperatures. The study also showed that chitosan polymers are compatible with doxycycline and protect it from degradation at 4 °C. There is still some work needed to improve the mechanical characteristics of the films containing microparticles.

## 4. Materials and Methods

### 4.1. Materials

Chitosan, sodium thiosulfate, polyvinylpyrrolidone (povidone) k-30, doxycycline hyclate analytical standard (hyclate is an abbreviation for hydrochloride hemiethanolate hemihydrate), gelatin type-A and crude mucin type-II were obtained from Sigma-Aldrich Chemie GmbH (Steinheim, Germany). Doxycycline hyclate was obtained from Hovione (Macau, China). Hydroxypropyl methylcellulose (K4M) was provided by Colorcon (Dartford, UK). *Tert*-butanol and disodium edetate were purchased from Riedel-de Haen (Seelze, Germany). Magnesium chloride hexahydrate was obtained from Merck (Darmstadt, Germany). Eudragit^®^ RS 12.5 was provided by Evonik Industries (Essen, Germany). Potassium dihydrogen phosphate was obtained from Fluka (Sigma-Aldrich Chemie GmbH, Steinheim, Germany) and β-cyclodextrin (Kleptose^®^) was provided by Roquette pharmaceuticals (Lestrem, France).

### 4.2. Doxycycline and β-Cyclodextrin Complexation

The doxycycline and β-cyclodextrin complexes were manufactured using three different methods, described in Patlolla et al. [17]. They are: (1) the physical mixture method [17,20,21]; (2) the kneading complexation method [17,21]; and (3) the freeze-drying method [20], where doxycycline and the β-cyclodextrin complex were added in 1:4 ratio to the deionized water and sonicated. The solution was frozen at −40 °C and then freeze dried. The complex obtained was sieved #100.

### 4.3. Preparation of Microparticles

Six different complexes were made as follows: (1) doxycycline in 2% *w/v* HPMC solution; (2) doxycycline in 2% *w/v* HPMC solution with 0.25% Eudragit^®^ RS 12.5; (3) doxycycline and β-cyclodextrin freeze-dried complex in 2% *w/v* HPMC solution; (4) doxycycline and β-cyclodextrin freeze-dried complex in 2% *w/v* HPMC solution with 0.25% Eudragit^®^ RS 12.5; (5) doxycycline, β-cyclodextrin, sodium thiosulfate and MgCl_2_ freeze-dried complex in 2% *w/v* HPMC; and (6) doxycycline, β-cyclodextrin, sodium thiosulfate and MgCl_2_ freeze-dried complex in 2% *w/v* HPMC with 0.25% Eudragit^®^ RS 12.5.

Next, 300 mL of the prepared polymeric solutions for each complex was spray dried using the Buchi spray dryer (BÜCHI 190 mini spray dryer). The heating control was set so that the inlet temperature was adjusted at 185 °C and outlet temperature was 125 °C. The feed was sprayed through a nozzle of 0.7 mm with a rate of 2.0 mL/min by a peristaltic pump. The pneumatic nozzle cleaning unit was attached to the nozzle and was switched on to clean all blockages arising from high viscous polymeric solutions. The conditions were similar for all the batches and the drying air rate was maintained at the highest level. A compressor (JUN-AIR) was attached to the instrument and the pressure was maintained at 5.6 bar. The microparticles were collected from the product collection flask and the walls of the cyclone chamber.

### 4.4. Preparation of Buccal Films

The buccal films were manufactured by the means of solvent casting method [22]. Chitosan 1% *w/w* was made by dissolving it in 1% acetic acid. Gelatin 2% *w/v* was made by dissolving in heated water on a hot plate. Povidone 2% *w/w* solutions were prepared in water. Each film was made in a 50 g batch, containing: 30 g of 1% chitosan, 11.25 g of 2% gelatin, 5 g of 2% povidone, 2.5 g of glycerin and 1.25 g of 12.5% Eudragit^®^ RS 12.5. Film 1 contained 27.7 mg doxycycline; Film 2 contained 174 mg doxycycline and β-cyclodextrin using microparticle method 3, described above; and Film 3 contained 232 mg doxycycline and β-cyclodextrin using microparticle method 6, described above. The doxycycline or microparticles were added to give 1 mg/cm^2^ concentration of doxycycline in the films.

The film solutions were centrifuged for 10 min before being cast into Petri dishes and allowed to dry in an oven at 40 °C until dry. The films were cut into required shapes and each was stored at 4 °C (refrigerator) and 25 °C (Newtronic humidity chamber) in a glass desiccator.

### 4.5. Analysis

#### 4.5.1. Fourier-Transform Infrared Spectroscopy (FTIR)

The FTIR spectra for the pure doxycycline, microparticles, buccal films and the excipients were recorded between wavenumbers 4000 and 400 cm^−1^, using the Nicolet iZ10 MX (Thermo Scientific, Waltham, MA, USA) and the software used was Omnic (Thermo Fisher Scientific, Waltham, MA, USA).

#### 4.5.2. Differential Scanning Calorimetry (DSC)

The thermograms for the doxycycline, microparticles, buccal films and excipients were recorded using the Netzsch Polyma DSC 214 instrument. A pierced aluminum pan was used as reference and approximately 2 mg of sample was placed into aluminum pans and the lids were sealed and the pans were heated at 10 °C/min with nitrogen gas (purge gas) at 20 mL/min. The temperatures were recorded between 25 °C and 350 °C.

### 4.6. Mucoadhesion Study of Buccal Films

The work of mucoadhesion and peak detachment force of the films were measured using the Texture Analyzer (TA-XT2i, Stable Microsystems, Godalming, UK) by slight modification of the method described by Skulason et al. [23]. A film sample (2 × 2 cm^2^) was attached to the floor of the instrument using a double adhesive tape. An artificial membrane, Duoderm, applied with a thin layer of artificial mucus (17% crude mucin type II, adjusted to pH 6.0) was adhered to the probe (P/10) to simulate the oral mucosa [23]. The pre-test, test and post-test speed of the probe was set at 0.1 mm/s. The contact time of the probe attached to the artificial mucus membrane with the film surface was set at 90 s to enable the mucoadhesive bonds and then probe and the contact force was set at 0.981 N. The trigger force was 0.010 N. The probe travel distance after the test was set at 10 mm. The peak detachment force was obtained from the force-time plot (F-T) and the work of mucoadhesion in mJ/cm^2^ was calculated by converting the plot to F-distance (Nmm).

### 4.7. Tensile Strengths and Elasticity

The mechanical properties of the films such as the tensile strength and the elasticity were evaluated using the Texture Analyzer (Stable Microsystems, Godalming, UK) with load cell 5 kg. The films were cut into 3 × 0.5 cm strips and attached to the “A/TG tensile grips” probe. The Texture Analyzer was operated in “Tension” mode with 5 s between two separation cycles. The probe, pre-test and post-test speeds were set at 0.1 mm/s and the test speed was 0.5 mm/s. The separation distance was set at 15.0 mm and the trigger force of the load cell was set at 0.010 N. The units were set as follows: force as Newtons (N) and distance as millimeters (mm). The elasticity was obtained in mm and converted to percentages. Each film was measured in triplicate. The tensile strength was calculated using Equation (1) [24] and the elongation at break using Equation (2) [24]:

(1) $$Tensile\;strength=\frac{breaking\;force}{cross\;sectional\;area\;of\;sample}$$

(2) $$Elongation\;at\;break\;\left(\%\right)=\frac{increase\;in\;length\;at\;breaking\;point}{original\;length}\times 100$$

### 4.8. Swelling Study of Buccal Films

The swelling of the films was performed by slight modification of the method described by Rana et al. [25]. The film samples of 1.5 × 1.5 cm^2^ were weighed and placed on a circular mesh and immersed in the swelling medium that consisted of a simulated saliva solution. At regular intervals, the mesh with film sample was sampled, dried and their weights noted. The degree of swelling (*SI*) was calculated using Equation (3):

(3) $$SI=\frac{W_{t}-W_{0}}{W_{0}}\times 100$$

where *W*_0_ is the weight of the film at time 0 and *W_t_* is the weight at time *t*. The simulated saliva solution contained 2.38 g/L disodium hydrogen phosphate, 0.19 g/L potassium dihydrogen phosphate and 8 g/L sodium chloride, where pH was adjusted to 6.75 using phosphoric acid [26].

### 4.9. pH, Film Thickness and Stability Studies

The pH of the films was measured as described by Bahri et al. [24], where 1.5 cm^2^ strip was added to a beaker containing 5 mL water and allowed to swell for 1 h. The pH was measured by bringing the pH electrode to the surface of the swollen film [27]. The thickness of the films was measured using a digital Vernier caliper (digiMAX, digital caliper).

The stability of doxycycline, alone or in different formulations, was studied at 4, 25 and 40 °C. The stability of films was studied at 4 and 25 °C. The data were recorded regularly for up to 6 months.

The microparticles were stored in tightly sealable amber bottles at 4 °C (refrigerator), 25 °C (Newtronic humidity chamber) and 40 °C (Venticell oven). The films were stored in a self-sealable bag, which was then placed in a desiccator stored at 4 and 25 °C.

### 4.10. Microparticles Dissolution Study and In Vitro Drug Release from Buccal Films

The microparticles dissolution study was carried out using the USP dissolution apparatus (RC-6D Dissolution Tester, Vanguard Pharmaceutical machinery INC, Spring, TX, USA) by slight modification of the method described by Hazra et al. [28]. First, 20 mg of each sample was weighed and suspended in 250 mL of 0.05 M dihydrogen phosphate (pH 6.75) at 37 °C, and the paddle rotation was set at 100 rpm. For the study of the release from buccal films, a strip of 1.5 × 1.5 mm was cut and sandwiched between the mesh and the bottom plate, so the mesh was exposed to the paddle. At regular intervals, 2.0 mL samples were collected and replaced with the fresh medium. All samples were diluted using 0.01 M HCl solution before the HPLC injection.

### 4.11. HPLC Method

The HPLC method was in accordance with the European Pharmacopoeia [29]. The HPLC instrumentation used was a Dionex system (Dionex Softron GmbH, Germering, Germany) comprising auto-sampler ASI-100, UV-visible detector, p680 pump with inbuilt DG-1210 degasser. The stationary phase was composed of PLRP-S styrene-divinyl benzene copolymer column (Agilent) with 250 mm × 4.6 mm and pore size 8 µm. The column was maintained at 60 °C with flowrate of 1 mL/min. The standards and samples were manufactured with 0.01 N HCl solution. The injection volume was 20 µL. The standard curve was obtained with five serial dilutions. The samples were diluted with 0.01 N HCl solution and concentration was adjusted in the mid-range of the calibration curve.

### 4.12. Microscopy

The mean microparticle size was analyzed using oil immersion technique using Olympus BH-2 (Olympus America, Norfolk, VA, USA) microscope equipped with a scale bar.

### 4.13. Statistics

The statistical analysis was carried out by the mean of R-Studio (RStudio, Inc., Boston, MA, USA). The statistical significance was calculated using the *t*-test, one-way analysis of variance (ANOVA) and the significance threshold was *p* < 0.05.

## 5. Conclusions

The main aim of the study was to evaluate the stability of doxycycline in buccal formulation at 25 °C. Microparticles were able to improve the stability of doxycycline as well as precomplexed doxycycline. β-cyclodextrin was found to improve the stability in solid form. Addition of antioxidants, chelating agents and complexing agents, also improved the stability. The microparticles, however, decreased several physical parameters and characteristics of the formulation that may affect the residence time and they also decreased the tensile strengths. The addition of precomplexed doxycycline to develop microparticles is novel and was found to be efficient in stabilizing doxycycline at higher temperatures (25 °C).
